# Ultrasound for Drug Synthesis: A Green Approach

**DOI:** 10.3390/ph13020023

**Published:** 2020-01-31

**Authors:** Micheline Draye, Gregory Chatel, Romain Duwald

**Affiliations:** Université Savoie Mont Blanc—LCME, F-73000 Chambéry, France; gregory.chatel@univ-smb.fr (G.C.); romain_duwald@yahoo.fr (R.D.)

**Keywords:** ultrasound, sustainable drugs synthesis, sonochemistry, green oxidations, green reductions, enzymatic transformations

## Abstract

This last century, the development of new medicinal molecules represents a real breakthrough in terms of humans and animal life expectancy and quality of life. However, this success is tainted by negative environmental consequences. Indeed, the synthesis of drug candidates requires the use of many chemicals, solvents, and processes that are very hazardous, toxic, energy consuming, expensive, and generates a large amount of waste. Many large pharmaceutical companies have thus moved to using green chemistry practices for drug discovery, development, and manufacturing. One of them is the use of energy-efficient activation techniques, such as ultrasound. This review summarizes the latest most representative works published on the use of ultrasound for sustainable bioactive molecules synthesis.

## 1. Introduction

**Figure 1:**
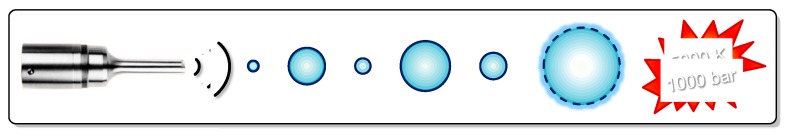
Growth and collapse of microsized bubbles in a liquid submitted to ultrasonic irradiation.

The 20th century has been strongly marked by the development of the chemical industry, which has been the cause of considerable improvements in the living conditions of its contemporaries. One of its greatest successes is the development of organic medicinal molecules that still play an important role today in the life of humans [1] and animals. Unfortunately, the synthesis of drug candidates requires the use of many chemicals, solvents, and processes that are very hazardous, toxic, energy consuming, expensive, and generate a large amount of waste. Chemical and pharmaceutical industries are then faced with serious environmental problems. Thus, in the past decade, many large pharmaceutical companies have moved toward using green chemistry practices for drug discovery, development, and manufacturing [2]. Indeed, green chemistry is a new concept developed in the field of chemistry and chemical technology to guide the development of more sustainable synthetic methodologies and chemical processes, and to accommodate conventional existing procedures by making them both economically and environmentally acceptable. This is made possible by preventing the use of volatile and toxic solvents; using benign and alternative chemicals, such as non-toxic bio-sourced feed-stocks; minimizing chemical waste; and/or reducing energy consumption by using more energy-efficient activation techniques, such as ultrasound. Indeed, ultrasound is known to improve the reactivity and kinetics of some processes through the formation, growth, and collapse of micro-sized bubbles when a pressure wave of adequate intensity propagates through an elastic medium [3] (Figure 1). When imploding, these bubbles release a large amount of energy and induce localized extreme conditions of temperature (up to 5000 K) and pressure (up to 1000 bar), leading to high-energy free radical reactions and also interesting physical effects [4,5], making it possible to perform reactions in a heterogeneous phase, in water, with crude reagents [6], and enabling obtaining cleaner products with few or no by-products, while saving energy [7]. Thus, the catalyst activity may be enhanced under ultrasonic irradiation (20 < f < 80 kHz) through the improvement of the mass transfer between the liquid and its surface, as well as an increase of its specific area by a reduction of its particle size, and an acceleration of suspended particle motion, resulting in shock waves and microstreaming.

## 2. Ultrasound and Green Solvents: A Smart Combination

Often used in large volumes, solvents have received much attention in recent years in parallel with the development of green chemistry [8]. Indeed, even if the solvent is not directly responsible for the composition of the product of the reaction, its role is often crucial for its progress. Despite the success of the modern pharmaceutical industry, waste minimization and sustainability are still largely unheard of. As an example, according to a life cycle study of waste produced from some facilities of GlaxoSmithKline, 80% of their waste is solvent-related [9,10,11]. Thus, the use of a sustainable solvent that allows for whole reactants to be dissolved, the reaction to proceed, and the catalysts to be recycled is of prime interest alongside the health and safety of workers, as well as the environment. In addition, new legislations, such as REACH (Registration, Evaluation, Authorization and restriction of CHemicals) in Europe, are now affecting the import and usage of a wide range of chemicals [12].

In this context, several solutions are available, such as the reduction of the use of solvents [13]; the use of benign solvents like water; the use of negligible vapor pressure solvents, such as ionic liquids at room temperature; the use of bio-based solvents; or not using any solvent at all [14,15].

### 2.1. Drug Synthesis in Water

As Sheldon said in 1996: “if a solvent (diluent) is needed it should preferably be water” [16]. Indeed, water is a cheap, non-flammable, abundantly available, and ecofriendly solvent. In addition, through its unique physico-chemical properties, water is able to increase the rates and selectivities of various chemical reactions via hydrophobic effects [17,18]. In addition, water favors cavitation, especially between 318 and 343 K, making this solvent ideal for sonochemistry [15,19]. Thus, a common use of ultrasonic irradiation and aqueous media should be a smart combination for the development of environmentally sustainable protocols. In 1927, Richards and Loomis published the first experiments of sonochemistry in water, describing the acceleration of chemical reactions using ultrasonic irradiation [20]. Since a large number of papers have been published on the combination of ultrasound and water for performing organic reactions, we report in this review some results on the combination of ultrasound and water for the synthesis of biologically active molecules under catalyst-free conditions. These papers are representative in that sense that none of them cautiously describe the ultrasonic equipment and practical working parameters used; these parameters are key factors affecting the ultrasound efficiency and its impact on the media it gets through. This jeopardizes the reproducibility and comparability of the results, and the possibility of a rational explanation of its influence. However, even if reaction conditions and hardware apparatus are not fully detailed, ultrasound effects are clearly highlighted in those articles.

#### Ultrasound-Assisted Synthesis of Biologically Active Heterocycles in Water Under Catalyst-Free Conditions

Heterocycles are a very important class of compounds that have a wide range of applications, especially for the manufacture of pharmaceuticals, agrochemicals, and veterinary products [21]. Due to their importance, several papers were found in the literature that describe protocols for their synthesis that are highly questionable from a sustainable development perspective.

**Scheme 1:**
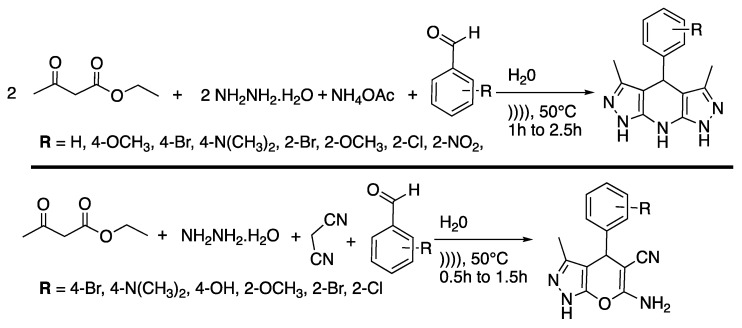
Synthesis of tetrahydropyrazolopyridines and pyranopyrazoles via a catalyst-free, four-component procedure in water under ultrasonic irradiation. The ultrasonic cleaning bath used P_output_ = 250 W and f = 40 kHz. Adapted from Shabalala et al. [24].

Pyrazoles and their derivates are very important biologically active moistures molecules that are contained in drugs, such as Celebrex® [22] and Viagra® [23]. Several protocols are available for their synthesis but they often suffer from a lack of selectivity, low yields, or by-product formation, or they require high temperatures, high reaction times, toxic solvents, or expensive reagents. Thus, Shabalala et al. [24] reported a simple, catalyst-free green synthetic protocol for the one-pot synthesis of pyrazoles in water under the ultrasonic irradiation of a simple bath cleaner (Scheme 1).

Compared to silent conditions, the tetrahydropyrazolopyridines were obtained selectively two to four times faster under ultrasonic irradiation, in excellent yields higher than 90%. Under ultrasonic irradiation, pyranopyrazoles were obtained selectively 2.5 to 6 times faster, also in excellent yields higher than 90%. In addition, under those conditions even aromatic spatially hindered aldehydes react in good yields, making this method excellent for the synthesis of tetrahydropyrazolopyridines and pyranopyrazoles. The results tend then to prove that ultrasound increases the kinetic and yield of the reaction, but the authors just suggest that this observation may be due cavitation.

**Scheme 2:**
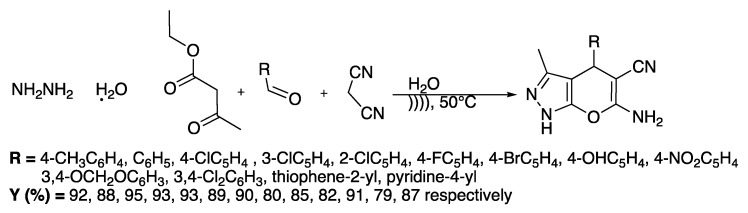
Ultrasound-assisted synthesis of dihydropyrano[2,3-c]pyrazole dervatives in water. The ultrasonic cleaning bath was thermostatically adjustable with f = 40 kHz and P_output_ = 250 W. Adapted from Zhou et al. [26].

In the same way, dihydropyrano[2,3-c]pyrazoles are very interesting for their biological properties such as autotaxin inhibition [25]. Zhou et al. [26] proposed a catalyst-free protocol for the synthesis of dihydropyrano[2,3-c]pyrazoles derivatives in water under ultrasonic irradiation (Scheme 2). The reaction was first finalized with hydrazine, ethyl acetoacetate, 4-methylbenzaldehyde, and malonitrile as model reagents.

A study of the effect of the solvent at 30 °C showed that the use of water led to the best yield (88%) in the shortest time (36 min). An increase of the temperature to 50 °C enabled improving the yield to 92% after 35 min of irradiation.

The method was then examined by reacting several substituted aryl or heteroaryle aldehydes, hydrazine, ethyl acetoacetate, and malonitrile in water at 50 °C under ultrasonic irradiation and the yields were compared to those obtained under silent conditions. Under ultrasonic irradiation, excellent results were obtained when the aromatic aldehyde was bearing electron-withdrawing or -donor substituents. Comparatively, under silent conditions, the yields were low and the reactions took a longer time. The authors attributed the beneficial effect of ultrasound to the solid heterogeneous systems to the acceleration of their dissolution and mass transfer improvement. To verify the mechanism of this one-pot, four-component, catalyst-free reaction, the authors performed three experiments under ultrasound and silent conditions. The results showed that the rate-determining step is the Michael addition of the carbanion of malonitrile to an intermediate issuing the condensation of the aromatic aldehyde with the tautomer of 3-methyl-5-pyrazolone; this step was completed in 25 min under ultrasonic irradiation, whereas 50 min was required under silent conditions.

**Scheme 3:**
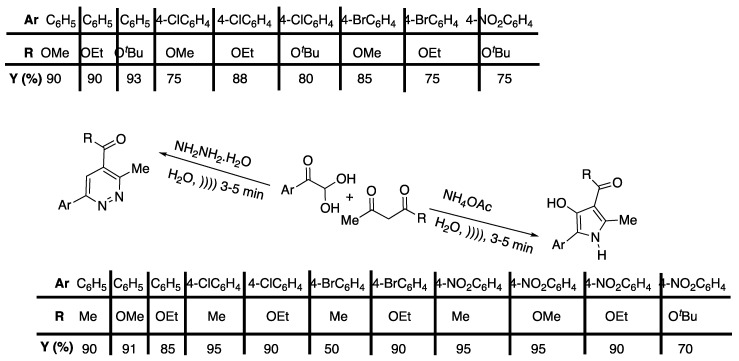
Ultrasound-promoted synthesis of pyridazines and 3-hydroxy-pyrrole-based derivatives in water and under ultrasonic irradiations. Adapted from Eftekhari-Sis and Vahdati-Khajeh [29].

Pyrroles and pyridazine are largely found in biologically and pharmaceutically active compounds, such as anti-tumor agents [27] or antidepressants [28]. In 2013, Eftekhari-Sis and Eftekhari-Sis and Vahdati-Khajeh [29] published a three-component, catalyst-free swift and efficient procedure for the synthesis of 5-aryl-4-hydroxy-2-methyl-1-*H*-pyrrole-3-carboxylic acid esters and 6-aryl-4-hydroxy-3-methyl-pyridazine-4-carboxylic acid esters using water as a solvent and under ultrasonic irradiation (Scheme 3).

After completion of the reaction, the products were isolated in high yields (50–90%) by simple filtration. A mechanism was proposed involving the in situ formation of an enamino ester and its attack onto the phenylglyoxal. Unfortunately, no information is given about the ultrasonic device, neither about the power nor the frequency used, making this experiment difficult to reproduce.

**Scheme 4:**
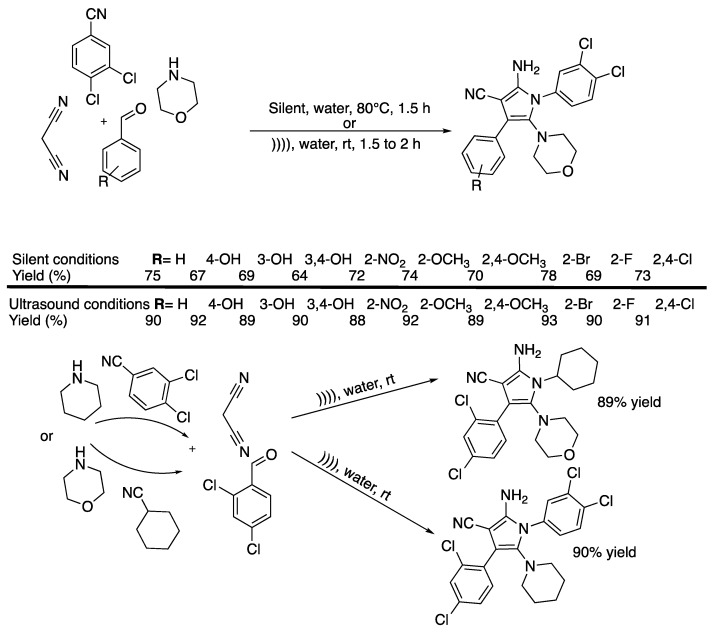
Catalyst-free, four-component synthesis of multi-substituted pyrroles under ultrasonic irradiation in water. An ultrasonic cleaning bath was used with f = 40 kHz and P_output_ = 250W. Adapted from Pagadala and Anugu [30].

A few years later, Pagadala and Anugu [30] published an ultrasound-promoted protocol for the synthesis of tetrasubstituted pyrrole derivatives in water under catalyst-free conditions using a simple bath cleaner (Scheme 4).

They finalized the reaction using malonitrile, benzaldehyde, morpholine, and 3,4-dichlorophenyl isocyanide as model reactants. They studied the effect of the solvent and the temperature, both under ultrasound and silent conditions. They observed that the reaction carried out at 80 °C under silent conditions in acetonitrile, ethanol, and water as solvents led to low to good yields, and the best result was obtained in water. Furthermore, the reaction did not take place at room temperature under silent conditions whatever the solvent. The authors then claimed that the same reaction performed in water under ultrasound irradiation at room temperature yielded the desired product selectively at a 90% yield. Furthermore, since the ultrasonic cleaner bath was not thermostated, the authors manually controlled the temperature to 25 ± 5 °C through the addition or removal of small amounts of water.

**Scheme 5:**
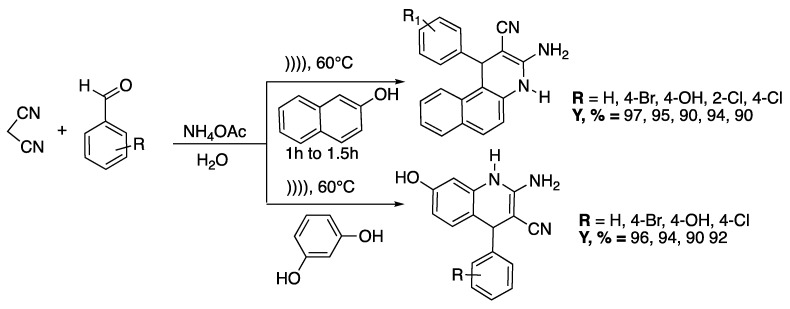
Catalyst-free, multi-component synthesis of dihydroquinolines under ultrasound irradiation in water. An ultrasonic bath was used with f = 40 kHz and P_output_ = 250 W. Adapted from Pagadala et al. [31].

Pagadala et al. developed [31] also an efficient and swift catalyst-free, multi-component protocol for the condensation of malononitrile with 2-naphthol or resorcinol, various aldehydes, and ammonium acetate. The reaction that was performed in aqueous medium under ultrasound irradiation at 60 °C led to the synthesis of a wide range of valuable dihydroquinolines in high yields (90–97%) (Scheme 5). Indeed, a quinoline scaffold is found in many natural products and pharmaceutically active substances [32].

Starting the model substrate (R = H) under ultrasonic irradiation and in ethanol as solvent, the reaction was completed in 3 h with a 75% yield instead of 5 h and 55% yield under silent conditions. Under ultrasonic irradiation with water as a solvent, the reaction was completed in 1 h with a 96% yield instead of 4 h and 80% yield under silent conditions. Various aldehydes were then subjected to the optimized conditions, leading to solid single products without by-products formation in a short time, in high yields, and easily purified by recrystallization. The authors classified this process as a heterogeneous sonochemical process.

**Scheme 6:**
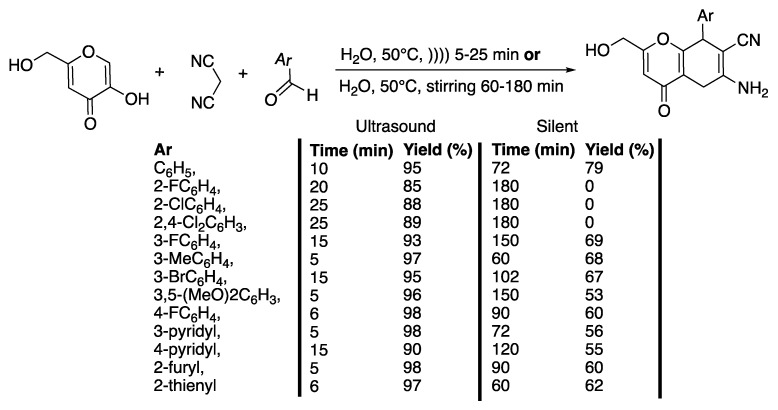
Catalyst-free synthesis of 2-amino-4,8-dihydropyranol[3,2-b]pyran-3-carbonitriles in water under ultrasonic irradiation. The ultrasonic cleaning bath used f = 40 kHz, P_output_ = 160 W, water, and T = 50 °C. Yields of isolated products are in %. Adapted from Banitaba et al. [34].

4-Aryl-4H-chromenes are potent apoptosis-inducing agents possessing vascular-disrupting activity and are studied as potential anti-cancer agents [33]. In that context, Banitaba et al. [34] developed a catalyst-free, combinatorial protocol in water as a solvent and under ultrasonic irradiation for the synthesis of polysubstituted 2-amino-4*H*-pyran-3-carbonitrile derivatives (Scheme 6).

With phenyl as a substituent and at an input ultrasonic power of 80 W, the authors showed that water is the best solvent, allowing for an 88% isolated yield in 20 min at 50 °C. Under silent conditions, at 50 °C, and in the same time, the yield did not go beyond 75%. In water and under ultrasonic irradiation, as well as under silent conditions, an increase of the temperature from 50 °C to 70 °C did not improve the yield. At 70 °C and under ultrasonic irradiation, the yield decreased from 88 to 80%. The authors ascribed this phenomenon to an increase of the content of vapor in the cavitating bubbles leading to a decrease of their values of maximum temperature and pressure. Under ultrasonic irradiation at 30 °C, a polar protic solvent, such as ethanol or methanol, afforded moderate yields of 70% and 60% of the desired products in longer reaction times of 50 and 55 min, respectively. In contrast, in acetonitrile, dioxane, and dichloromethane and at a lower temperature of 30 °C, the desired products were obtained at 45%, 45%, and 35% yields, respectively. When performed under silent conditions and whatever the experimental conditions of the solvent and reaction time, the yields were lower, confirming then the efficiency of ultrasound irradiation for the reaction. An increase of the input power from 40 to 160 W, and thus of the ultrasound intensity, leads to an increase of the yield and a decrease of the reaction time from 25 to 10 min. Then, for 10 min of ultrasonic irradiation, an increase of the power from 160 to 200 W decreases the yield from 95 to 90% due to a scattering effect of the gas bubbles on the sound waves. Several heteroaryl or substituted aldehydes were then examined under ultrasonic irradiation and under silent conditions. The results show that under silent conditions, a longer time is required to obtain a lower yield in 2-amino-4,8-dihydropyranol[3,2-b]pyran-3-carbonitriles derivatives (Scheme 6), probably due to a problem of solubility of the aldehydes; under ultrasonic irradiation, the yields are excellent for 5 to 25 min of irradiation, depending on the substitution of the starting aldehyde, because of a beneficial effect of ultrasound on solubility behavior. The authors then proposed a mechanism involving a Knoevenagel condensation, a Michael addition, and a cycloaddition, which might be initiated through two pathways. In both cases, ultrasonic irradiation may favor the establishment of an interaction in the dipolar transition states, facilitating the cyclization and dehydration step that is critical in the type of multi-component reactions. As a conclusion, this method presents many advantages, such as shorter reaction times, no need for a catalyst, excellent yields, and environmental friendliness.

**Scheme 7:**
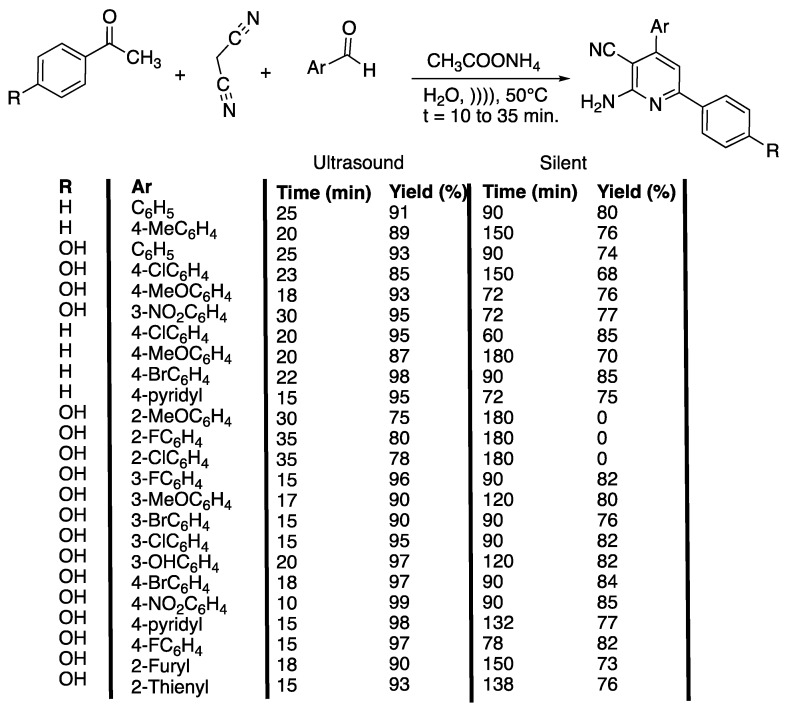
Catalyst-free method for the one-pot synthesis of 2-amino-4,6 diphenylnicotinitriles in water and under ultrasonic irradiation. The ultrasonic cleaner bath used f = 40 kHz, P_output_ = 250 W, water, and T = 50 °C. Adapted from Safari et al. [36].

2-Amino-pyridine derivatives have been identified as potential derivatives for the treatment of prion disease [35], as IKK-b inhibitors, A2A adenosine receptors antagonists, and potent inhibitors of HIV-1 integrase [36], and anti-tubercular agents [37]. Despite the large number of procedures available for the synthesis of 2-amino-3-cyanopyridines, the most common use multiple steps, long reaction times, the use of toxic solvents and a catalyst, high temperatures, and lead to modest yields of the product [38]. Safari et al. [36] also developed a catalyst-free method for the one-pot synthesis of 2-amino-4,6-diphenylnicotinitriles in water and under ultrasonic irradiation (Scheme 7).

The authors first optimized some parameters, such as the solvent nature and temperature under ultrasonic irradiation, by reacting malonitrile with acetophenone, benzaldehyde, and ammonium acetate. In water at 30 °C, the reaction proceeded fast (45 min) in a high yield (83%). In polar protic solvents, such as ethanol and methanol and at 30 °C, the reaction proceeded slower (70 and 75 min) with lower yields (70 and 65%). At 30 °C, in acetonitrile, in THF, in CH_2_Cl_2_, and in solvent free conditions, the yields were even lower (35%, 40%, 30%, and 60%, respectively), and the reaction took even longer (150 min, 120 min, 160 min, and 110 min, respectively). In water, an increase of the temperature from 30 °C to 50 °C was shown to lead to an increase of the yield from 83% to 91% in 20 min less. An additional increase of the temperature was not beneficial because of the loss of ammonia. When performed under silent conditions, the reaction took a longer time and proceeded with lower yields. The best conditions were applied to several substituted aryl or heteroaryl aldehydes, acetophenones, malonitrile, and ammonium acetate in water at 50 °C under ultrasonic irradiation, and the results were compared to those obtained under silent conditions. As a general manner, the yields and times of reaction are improved by the use of ultrasound, without the need for a catalyst. In addition, the use of ultrasonic irradiation enables 2-methoxy, 2-chloro, and 2-fluoro spatially hindered aldehydes to react and to give the desired products with good yields (75%, 80%, and 78%, respectively) and short times (30, 35, and 35 min, respectively) (Scheme 7). The authors proposed a mechanism that, thanks to cavitation phenomenon, involves dipolar transition states, facilitating the production of ammonia, cyclization, and dehydration step that is usually critical in this type of multi-component reaction. Furthermore, electron-withdrawing groups on aromatic aldehydes were found to facilitate the nucleophilic addition of acetophenone to arylidinemalononitrie or malonitrile, depending on the pathway followed. In contrast, the nature of the substituent on the aromatic acetophenone ring did not shown any obvious effect. In summary, an efficient method was found for the catalyst-free synthesis of 2-amino-4,6-diphenylnicotinonitrile ring systems using ultrasonic irradiation in water.

**Scheme 8:**
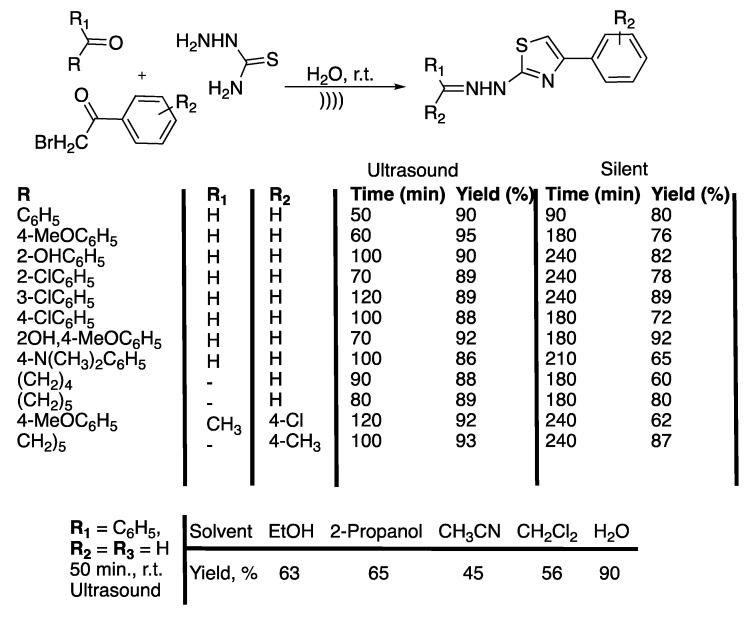
One-pot, three-component, catalyst-free synthesis of N-(4-arylthiazol-2-yl) hydrazones in water under ultrasonic irradiation. The ultrasonic cleaner bath used f = 25 kHz, P_input_ = 250 W, and T = 25 °C. Adapted from Zhang et al. [42].

Thiazole derivatives of Schiff bases are well known for their pharmacological properties [39], where among them, hydrazothiazoles have attracted extensive interest because of their better therapeutic effects [40]. Unfortunately, their two-step synthesis often requires long reaction times, the use of toxic organic solvents, and produces only moderate yields [41]. In 2012, Ahng et al. [42] described a one-pot, three-component, catalyst-free reaction for the synthesis of N-(4-arylthiazol-2-yl)hydrazones in water under ultrasonic irradiation (Scheme 8). To study the effect of the solvent, they condensed benzaldehyde, thiosemicarbazide, and phenacyl bromide, which were used as model reagents. The reaction in water was completed in 50 min with a 90% yield, whereas in the same time but in ethanol, 2-propanol, acetonitrile, or dichloromethane as solvents, the yields did not exceed 65%.

In water as a solvent, an increase of the temperature from 25 to 35 °C and then to 45 °C decreased the yield from 90% to 82% and 78% in 50 min, 40 min, and 40 min of ultrasonic irradiation, respectively. The authors then compared the results to those obtained via the two-step method under silent conditions. The desired product was obtained with a 55% yield in 130 min and therefore shown to be less efficient. The one-pot, three-component, catalyst-free reaction in water was then extended to several aromatic aldehydes or ketones and substituted phenacyl bromide under ultrasonic irradiation or silent conditions. The results show that, under ultrasound, both aldehydes substituted with electron-rich and electron poor functionalities led to N-(4-arylthiazol-2-yl)hydrazones with better yields (86 to 95%) and in shorter reaction times than under silent conditions. The authors concluded that the described protocol is easy, highly efficient, is low cost, and is environmentally friendly.

**Scheme 9:**
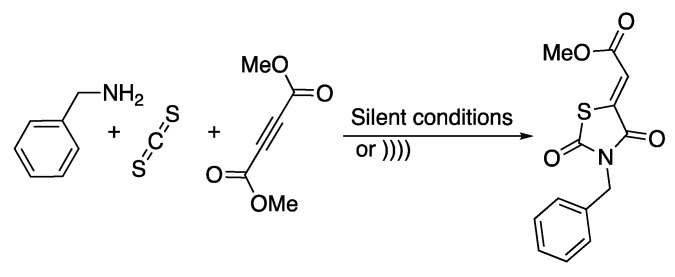
Synthesis of rhodanines derivatives in water under ultrasonic irradiation. The ultrasonic probe used f =20 kHz, P_output_ = 600 W, and t = 3 to 5 min. Adapted from Rostamnia and Lamei [44].

Rhodanines are known to possess fascinating properties and a very large spectrum of biological properties [43]. As a part of their ongoing research on the synthesis of rhodanine, Rostamnia and Lamei [44] developed a catalyst-free, one-pot protocol for the synthesis of rhodanine derivatives in water and under ultrasonic irradiation (Scheme 9).

Dimethyl acethylenedicarboxylate, carbone disulfide, and benzylamine were used as reagents of a model reaction that was carried out under ultrasonic irradiation and under silent conditions in various solvents. Whatever the solvent used, the reaction was completed in 3 min with a high yield (Table 1). The best yield was obtained in water under 3 min of ultrasonic irradiation. Under those conditions, decreasing the volume of water from 40 to 10 mL had a negligible effect.

The reaction was then extended to other amines and alkyne derivatives with water as the solvent, and good to excellent yields (86 to 94%) were observed in 3 to 5 min of ultrasonic irradiation. The authors confirmed that under ultrasonic irradiation, the mechanism proposed by Alizadeh et al. [45] under silent conditions took place.

**Scheme 10:**
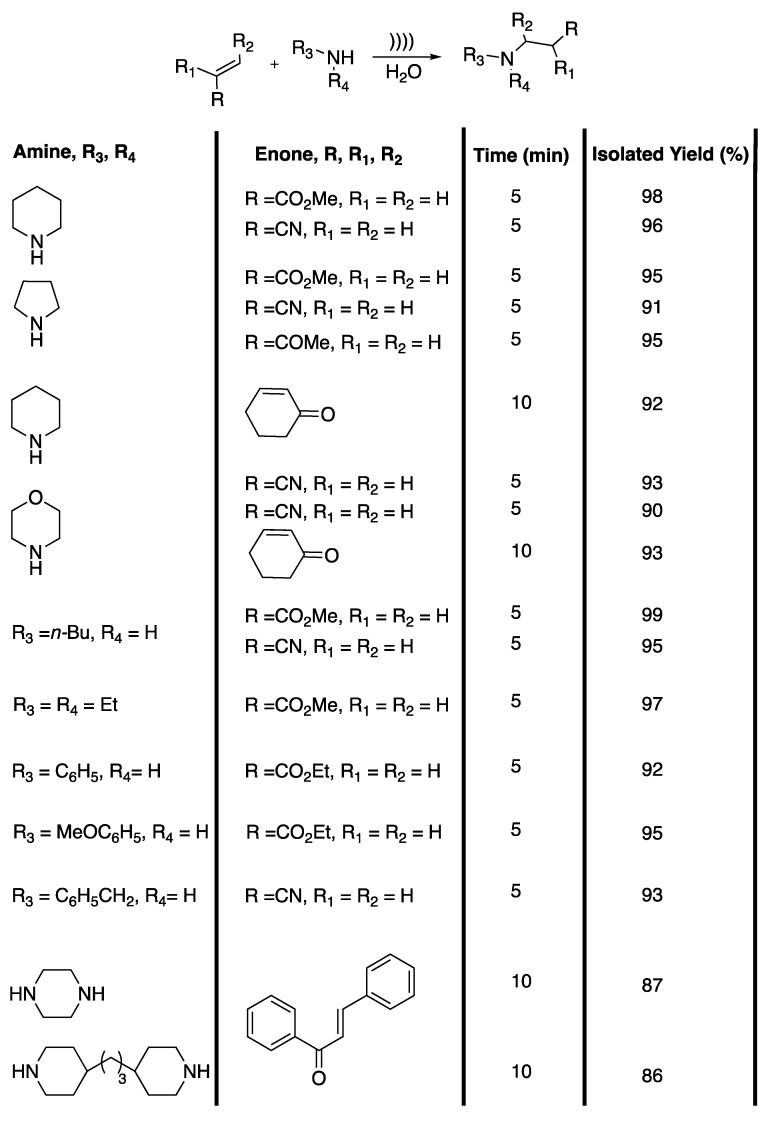
Catalyst-free, ultrasound-assisted aza-Michael reaction in water. The ultrasonic cleaner bath used f = 42 kHz, P_output_ = 135 W, and t = 5 to 10 min. Adapted from Bandyopadhyay et al. [47].

β-aminocarbonyl and β-amino acid derivatives are useful building blocks for the synthesis of biologically active substances and pharmaceuticals [46]. Unfortunately, the main methods described in the literature for obtaining them require costly and/or toxic catalysts, long reaction times, hazardous solvents, and lead to low yields after a difficult product isolation. In 2012, Bandyopadhyay et al. [47] developed a catalyst-free, ultrasound-assisted aza-Michael reaction in water (Scheme 10).

The authors first determined the most efficient solvent using a model reaction with piperidine and methyl acrylate as reactives. Moderate to excellent yields were obtained in polar protic solvents and the desired product was isolated with a 98% yield under 5 min of ultrasonic irradiation in water. In ethanol or methanol, 62% and 58% yields were obtained in 60 min of irradiation, whereas 93% yield was obtained in only 15 min of irradiation under solvent-free conditions. Moderate yields were obtained in polar and apolar aprotic solvents under 60 min of ultrasonic irradiation. In water and under silent conditions, a 96% yield was obtained in 30 min of stirring at room temperature. The ultrasound-induced reaction in water was then extended to various amines and unsaturated ketones, unsaturated nitriles, and unsaturated esters; excellent isolated yields of 86% to 98% were obtained in 10 to 5 min of ultrasonic irradiation with high regio- and chemo-selectivity, and without side products. Primary amines selectively led to mono addition products without bis addition occurring. The authors explained that the good yields observed were due to three phenomena: 1) the assistance of water as the solvent through hydrogen bond formation with the carbonyl group increasing the electrophilic character at the β-carbon of the unsaturated compounds; 2) hydrogen bond formation between the oxygen atom of water and the hydrogen atom of the amine, increasing the nucleophilic character of the *N*-atom of the amine; and 3) water acting as a pseudo-organic solvent at the temperature of the bubble implosion under ultrasonic irradiation. In summary, this efficient, catalyst-free, simple, and environmentally friendly procedure in water is a fine addition to the aza-Michael addition procedures for drug synthesis.

**Scheme 11:**
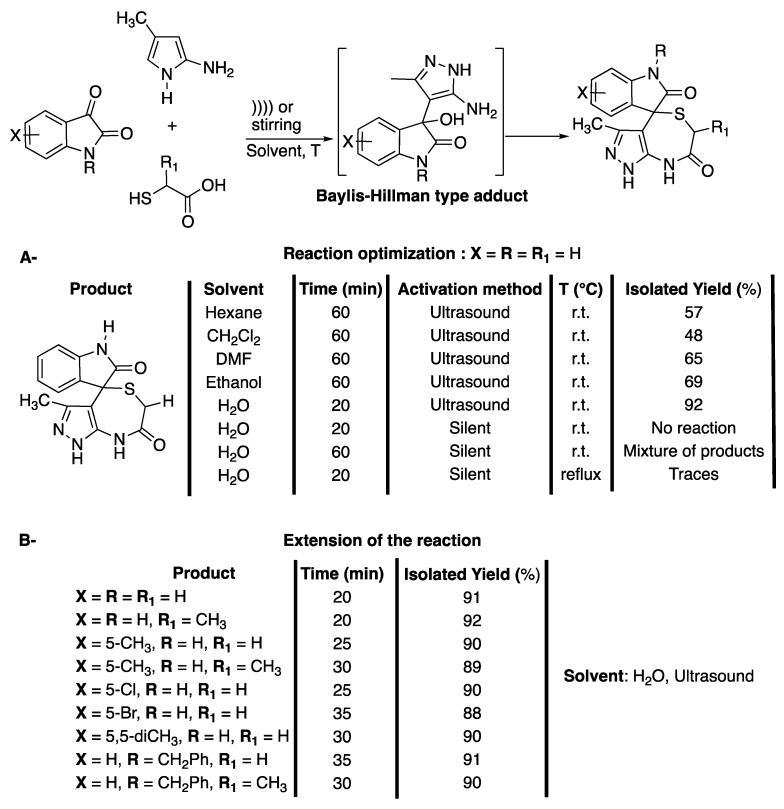
Synthesis of spiro[indole-3,4′-pyrazolo[3,4-e][1,4]thiazepines]. The ultrasonic probe used f = 20 kHz and P_output_ = 750 W. Adapted from Dandia et al. [53].

Thiazepine and diazepine derivates have been identified as important motifs due to their important biological and therapeutic activities. Benzothiazepines have been identified as calcium antagonists, leading to the development of Diltiazem for the treatment of diseases such as high blood pressure or angina [48]. Meanwhile, dibenzothiazepines have been widely used for antipsychotic drugs, such as quetiapine [49] or clothiapine [50], and tricyclic benzothiazepines have been identified as potential anti-drug-resistant cancer agents [51]. Despite their importance, most processes for their synthesis require harsh conditions, expensive reagents, high catalyst loading, corrosive or toxic reagents, and often lead to several side reactions [52]. In 2013, Dandia et al. [53] developed a three-component reaction under catalyst-free conditions and ultrasonic irradiation for the synthesis of spiro[indole-3,4′-pyrazolo[3,4-e][1,4]thiazepines] in water. The reaction was shown to be chemo-, regio-, and diastereo-selective, and the formation of the thiodiazepin seven-membered ring was conditioned by that of a Baylis-Hillman type adduct (Scheme 11). The diastereoselectivity of the reaction was confirmed using X-ray crystallography analysis.

The three-component reaction was first investigated at room temperature with a mixture of isatin, 5 amino-3-methylpyrazole, and α-mercaptoacetic acid (Scheme 11A) in water under ultrasonic irradiation. The efficiency of catalysts, such as *p*-TSA, AcOH, or InCl_3_ and CTABr, as a surfactant was studied. A maximum yield of 78% was obtained in 30 min of ultrasonic irradiation in the presence of CTABr. The effect of the solvent was then evaluated and yields of less than 50% were observed in hexane, dichloromethane, DMF, and ethanol, whereas 92% of the target product were obtained in water. The authors explained this result by the formation of hydrogen bonds between water and the 3-carbonyl of isatin, increasing the electrophilicity of its 3-carbon, and between water and thiol hydrogen. In addition, water hydrogen is suspected to work as a Brønsted acid, activating the Baylis-Hillman type adduct (Schrmr 11A). In addition, this heterogeneous reaction was shown to take advantage of the physical effects of ultrasound, such as the enhancement of mass transfer, micromixing, increase of the kinetic of dissolution, and an increase of the temperature. The reaction conditions were then extended to various substituted isatins and thioacids, affording good to excellent yields of a single isomer in 20 to 35 min of reaction, depending on the substrates used (Scheme 11B). The optimized reaction conditions were then successfully used for the synthesis of spiro[acenaphthylene- and spiro[piperidine-4,4′-pyrazolo[3,4-e][1,4]thiazepines], highlighting the potential of this protocol. Finally, their biological activities were explored for a potential use as anti-diabetic agents.

### 2.2. Drug Synthesis in Ionic Liquids

In the last three decades, ionic liquids (ILs) have been particularly investigated and used as reaction media [54,55,56,57]. This class of solvents has been defined as salts with low melting points (by definition, lower than 100 °C) [58] or no melting point, and presents several unique properties compared to traditional solvents, including negligible vapor pressure, high polarity, high chemical and electrochemical stabilities, etc. [59,60,61,62]. Their application as reaction media have been proposed for analytical chemistry [63], energy chemistry [64,65], biomass conversion [66,67,68], materials preparation [69,70,71], microextraction [72,73,74], electrochemistry [75,76,77,78], and organic chemistry [79]. Interestingly, their physico-chemical, thermal, and solvent properties are based on the nature of the ions that compose them, and more particularly, on the interactions between these ions. With the great number of ion combination possibilities, the tunability of these solvents is important and can lead to several different uses and applications. However, a lack of theoretical data about their properties represents a limiting drawback regarding guiding their use.

The synergistic combination of ILs with ultrasound has been increasingly investigated with several examples of applications reported in organic chemistry, extraction processes, in biomass valorization, materials preparation, electrochemistry, degradation of ILs, and many others [80]. The use of low ultrasound (US) frequencies is predominant in the literature, thanks to its ease of processing and its accessibility in laboratory conditions. Globally, the ILs/US combination favors the decrease of the reaction or preparation time and improves the reaction yield, selectivity, and/or quality of the products in comparison with silent conditions. Interestingly, some unexpected results were obtained under US/IL conditions, showing the potential for new synthesis pathways [81].

**Scheme 12:**
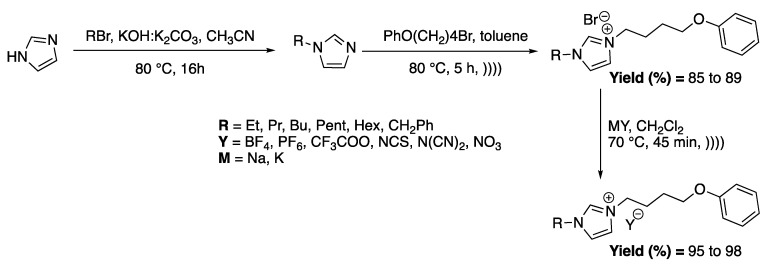
Synthesis of a variety of imidazolium-based ILs and anion metathesis using ultrasonic irradiation. Adapted from Messali et al. [82].

Messali et al. [82] directly studied the antimicrobial activity of 1-alkyl-3-(4-phenoxybutyl) imidazolium-based ILs derivatives prepared using ultrasound in a 25 kHz cleaning bath. Their activities are greatly affected by the alkyl chain length (Scheme 12).

For the quaternization step, the use of ultrasound allowed for obtaining better yields (average of 79% under conventional heating vs. 87% under ultrasound) and decreased the reaction time from 18 h under silent conditions to 5 h under ultrasound. The anion metathesis step was also improved under ultrasound, especially by decreasing the reaction time from 3 h to 45 min under ultrasonic conditions. Here, the effects of ultrasound were not studied, but the micromixing effects seem to be the main explanation for the improved results.

**Scheme 13:**
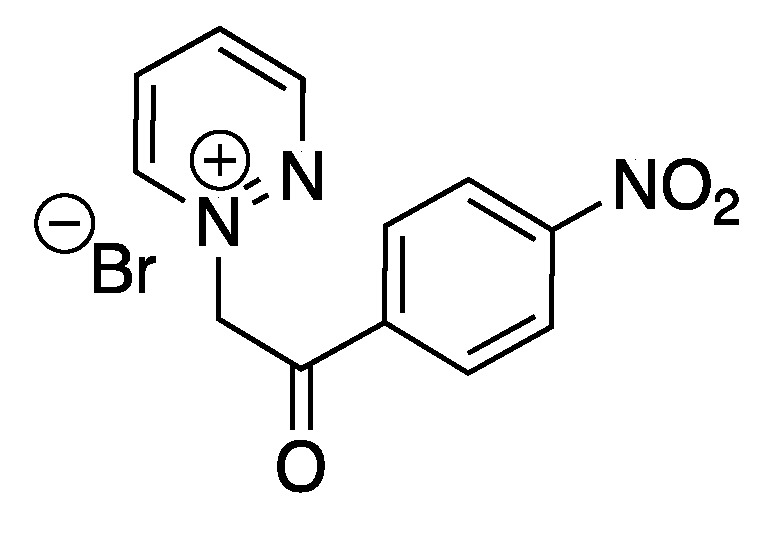
Bromide pyridazinium-based IL with the best antibacterial activity. Adapted from Messali et al. [83].

The same group also reported the ultrasound-assisted synthesis of new pyridazinium-based ILs, exhibiting good to moderate antibacterial activity [83]. Some of the tested ILs were also efficient in vitro against human breast adenocarcinoma, human colon carcinoma, and human hepatocellular carcinoma cell lines. An IL based on a bromide anion (Scheme 13) particularly exhibited promising antiproliferative effects, producing the lowest IC_50_ values among the tested ILs. The same reduction of reaction time was reported under ultrasound (25 kHz, cleaning bath) in comparison to silent conditions, without more investigation of the ultrasonic effects.

New pyridinium ILs with hydrozones tethering fluorinated counter anions were also prepared under ultrasound, showing novel inhibitors of fungal ergosterol biosynthesis [84]. The origin of this antifungal activity could be explained by the binding of the ILs to membrane ergosterol, leading to pore creation in fungal cell membranes, and then to the loss of intracellular content. Some further *in vivo* studies were suggested by the authors in order to test antifungal properties against *Candida* infections. In this example, US decreased the reaction time of IL preparation (50 kHz, 240 W, cleaning bath) by 3 to 5 times.

**Scheme 14:**
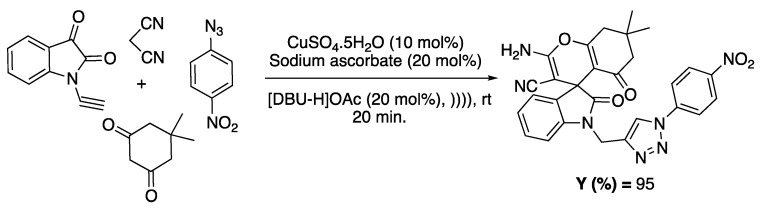
Four-component condensation for the synthesis of spiro-oxindole derivatives. Adapted from Sing et al. [85].

The combination involving ultrasound as an activation method and IL as a solvent/catalyst was also reported for the synthesis of compounds presenting antibacterial and antifungal activities. For example, Khurana and co-workers reported the synthesis of triazolyl and spirocyclic oxindole derivatives through a one-pot, four-component domino reaction of 1-(prop-2-ynyl)indoline-2,3-dione, malononitrile, cyclic 1,3-diketones, and various aryl azides. These syntheses were performed in DBU-based ILs ([DBU-H]OAc and [DBU-Bu]OH) under US irradiation (ultrasonic bath, 54 kHz, 300 W, 3 L capacity), favoring the construction of heterocycles (Scheme 14) [85].

The authors observed that the reaction was incomplete, even after 120 min, when performed in the absence of ultrasound, whereas a yield of 94% was obtained in only 15 min under ultrasonic activation. The exact role of ultrasound was not investigated in this study.

**Scheme 15:**
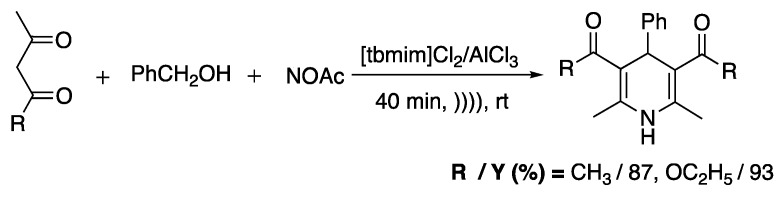
Synthesis of 1,4-DHP. Adapted from Reddy et al. [86].

Reddy et al. [86] used ionic liquids (3,3′-thionyl-bis-1,10-methylimidazolium chloroaluminate) and ultrasonic conditions (cleaner with a frequency of 40 kHz and a nominal power 100 W) for a one-pot three-component synthesis of 1,4-dihydropyridines. Authors noted that in the absence of the ionic liquid and without ultrasound irradiation, the yield of the reactions decreased with the increase in time. Here, even if the exact role of IL and US was not demonstrated, the effect on yield and reaction time was shown, and the existence of a stabilization effect should be explored (Scheme 15). 

Other studies reported the use of ILs as catalysts, supported [87] or added in aqueous media [88] in combination with ultrasound to produce pharmaceutical compounds. In the first case, a new poly(4-vinylpyridine)-supported acidic IL catalyst was synthesized from 4-vinylpyridine and 1,3-propanesultone after a polymerization and the addition of the heteropolyacid [87]. This heterogeneous catalyst was used to produce 2,3-dihydro-4(1H)-quinazolinones compounds through a cyclocondensation reaction of anthranilamide with aldehydes under activation (40 kHz, 250 W). In addition, the catalyst was recovered using filtration and reused six times without a significant loss of catalytic activity. The use of ultrasound significantly increased the kinetics of the reaction.

In the second case, ultrasound (frequency not specified) was used in the synthesis of new primary O-alkyl and O-aryl thiocarbamates through the reaction of aliphatic alcohols and phenols with thiocyanate salts in the present of *N*-methyl-2-pyrrolidonium hydrogen sulfate as a reusable catalyst [88]. The reaction was performed in aqueous media at room temperature under ultrasound irradiation. Compared with the conventional methods, this procedure led to more rapid reaction rates and better yields. This synthesis of primary O-alkyl and O-aryl thiocarbamates could be useful for drug discovery.

### 2.3. Drug Synthesis in Biosourced Solvents

Although most syntheses of pharmaceutical compounds involving ultrasound are carried out in water or without a solvent, some interesting examples have been reported in biosourced solvents [89,90,91]. For example, the microencapsulation of kraft lignin in the presence of olive oil, a lignin alkali solution, was optimized under ultrasound (microtip, frequency not specified, 40 s) creating an oil in water emulsion and a cross-linking of lignin at the water/oil interface [92]. In this example, lignin microcapsules incorporated and released coumarin-6. In vitro studies and confocal laser scanning microscopy analysis were used to confirm that lignin microcapsules were readily incorporated and their non-cytotoxicity was demonstrated on Chinese hamster ovary cells.

Glycerol was used in mixture with ethanol to extract some polyphenolic antioxidants from eggplant peels [93]. The advantages of glycerol-based processes are its lack of flammability and toxicity, its high boiling point, its abundance as a by-product of vegetable oils and biofuels, and its low cost [94]. In addition, the incorporation of glycerol extracts in pharmaceutical, cosmetic, and nutraceutical formulations is simple, since glycerol is extensively used as a constituent in such products. The physical effects of ultrasound irradiation (37 kHz, 140 W, 35 W·L^−1^, 90 min) are responsible for the increase of polyphenols in the extracts.

### 2.4. Solventless Drug Synthesis

Even if the pharmaceutical industry has undertaken a huge effort to replace toxic solvents, the ideal would be totally eliminating solvents that would comprise the product. Indeed, as Sheldon has said: “the best solvent is no solvent” [14]. Solvent-free processes should allow for eliminating a large amount of waste since solvents contribute to a large part of the total waste in the pharmaceutical industry [14]. In addition, solvent-free synthesis has the potential to be cost-saving and safe in some cases.

#### 2.4.1. Ultrasound-Assisted Synthesis of Biologically Active Heterocycles Under Solventless Conditions and with Organocatalysts

**Scheme 16:**
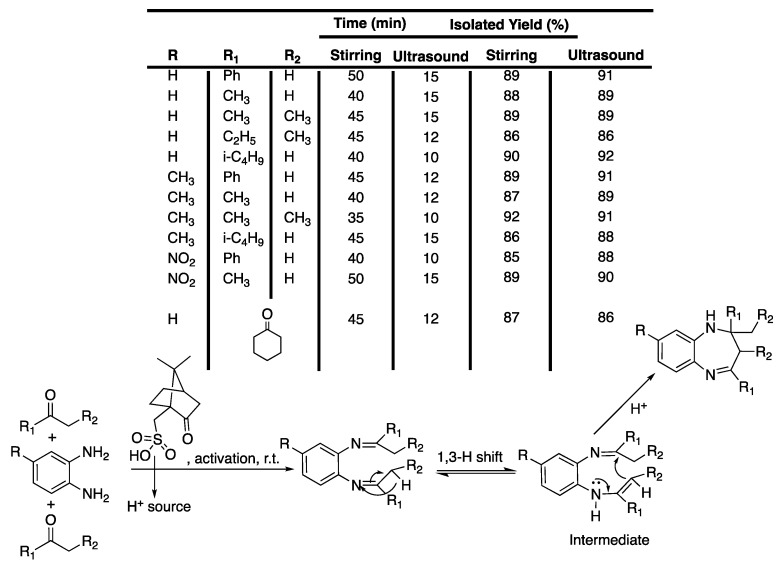
Mechanism for the solventless synthesis of 1,5-benzodiazepine derivatives involving camphor sulfuric acid. The ultrasonic bath used f = 35 kHz and P_input_ = 200 W. Adapted from Shinde et al. [96].

Benzodiazepines have been receiving great interest in the field of medicinal chemistry because of their various biological activities and their use as hypnotics, anxiolytics, anticonvulsants, and muscle relaxants [95]. The first report on the combined use of ultrasound irradiation and solvent-free conditions for the synthesis of 1,5-benzodiazepines was reported by Shinde et al. [96], who described works where camphor sulphonic acid was used as an eco-friendly organocatalyst. Indeed, homogeneous catalysts, such as H_2_SO_4_, HCl, or BF_3_, are corrosive, toxic, or volatile, and generate large amounts of waste [97], whereas heterogeneous ones present the advantage of being easily recovered and thus re-used. Among these, organocatalysts present additional advantages compared to metallic ones, such as a safer disposal and some flexibility in their design. In their work, they first reacted *o*-phenylenediamine with acetophenone as a model reaction [96]. The reaction was found to be impossible without a catalyst; thus various acid catalysts, such as boric acid, sulfamic acid, sulfanilic acid, EDTA·2Na salt in water, and pTSA, as well as some organocatalyts, such as L-proline and camphor sulphonic acid, were tested to activate the reaction. A maximum yield of 91% 2-methyl-2,4-diphenyl-2,3-dihydro-1H-1,5-benzodiazepine was obtained in the presence of 10% of camphor sulphonic acid under 15 min of ultrasonic irradiation and solvent-free conditions. The carbonyl group of the camphor sulphonic acid was proved to not react by performing a series of experiments without a second reactant bearing the ketone function. A mechanism was then proposed involving camphor sulphonic acid as a catalyst to form an intermediate, affording the final product after cyclization (Scheme 16). The synthetic methodology was then extended to various ketones giving similar or better yields under ultrasonic irradiation than those obtained under stirring (Scheme 16).

**Scheme 17:**
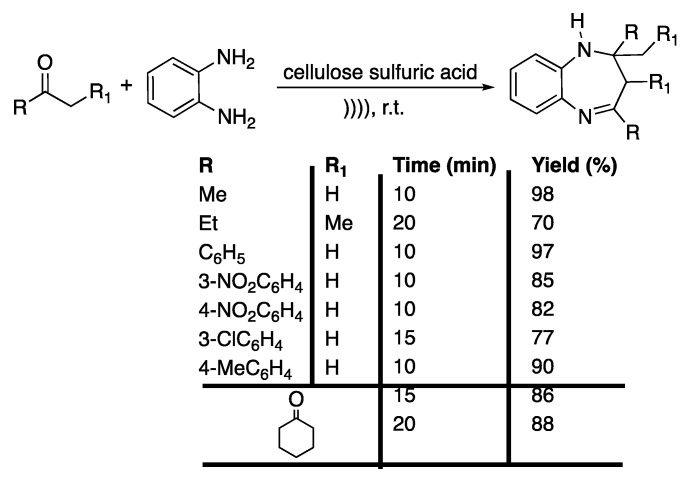
Ultrasound-assisted solventless synthesis of 1,5-benzodiazepine derivatives under catalyst-free conditions. The ultrasonic bath used f = 35 kHz. Adapted from Shelke et al. [98].

More recently, Shelke et al. [98] developed a solvent-free protocol for the ultrasound-assisted synthesis of 1,5-benzodiazepines derivatives with cellulose sulphuric acid (CSA) as a catalyst; owing to its non-toxicity and its bio-sourced origin, this is considered a good candidate. Using acetone and *o*-phenylenediamine as model reactives, the reaction was optimized in terms of the ratio of CSA, which exhibited a high catalytic activity. Next, the scope of the reaction was investigated by reacting a variety of ketones with *o*-phenylenediamine under the optimized conditions, providing the corresponding benzodiazepines in good to excellent yields in 10 to 20 min (Scheme 17). In addition, the recyclability of the catalyst was shown, where after three runs, the catalyst was still as efficient.

**Scheme 18:**
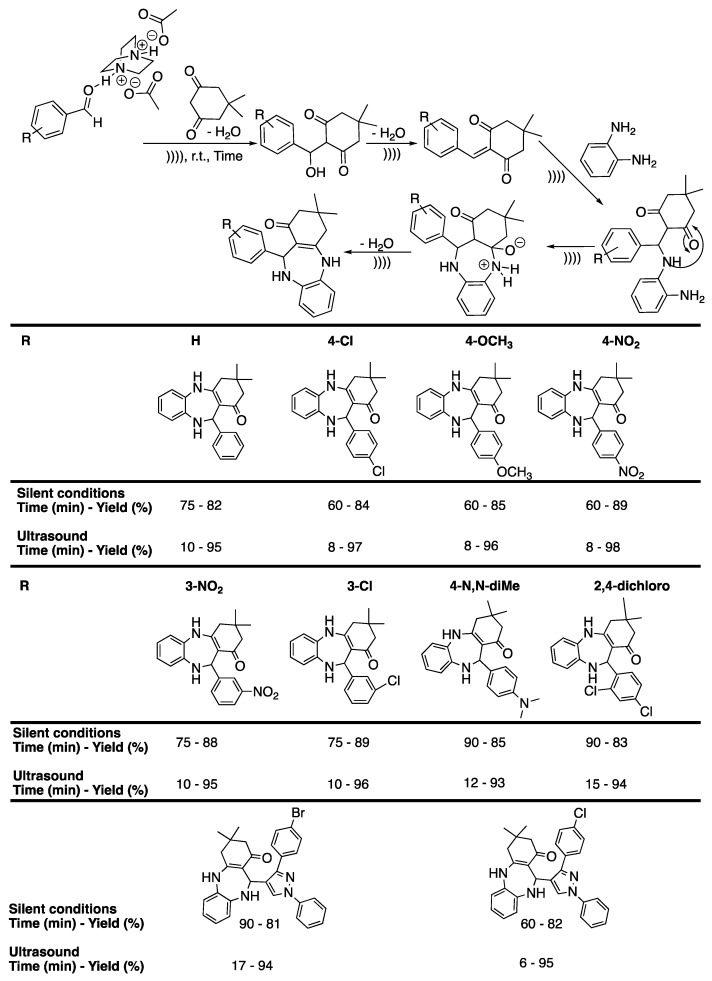
DABCO-triacetate-assisted synthesis of benzodiazepines under ultrasound in solvent-free conditions. Yields are isolated. The ultrasonic bath used f = 45 kHz and P_input_ = 305 W. Adapted from Fekri et al. [99].

Similarly, Fekri et al. [99] prepared 1,4-diazepin derivatives under ultrasonic irradiation and solventless conditions using an ionic liquid as a catalyst, synthesizing DABCO-diacetate under microwave irradiation. Various parameters were optimized by reacting 4-nitrobenzaldehyde, *o*-phenyldiamine, and dimedone. The reaction performed with butylmethylimidazolium bromide and hexafluoroborate did not proceed, whereas bis ionic liquid DABCO-diacetate was efficient for the synthesis of 3,3-dimethyl-11-phenyl-2,3,4,5,10,11-hexahydro-1H-dibenzo[b,e][1,4]diazepin-1-one. Using a catalyst loading of 0.5 mmol, 3,3-dimethyl-11-phenyl-2,3,4,5,10,11-hexahydro-1H-dibenzo[b,e][1,4]diazepin-1-one was obtained in 95% yield in 10 min under ultrasonic irradiation at room temperature, whereas 75 min of magnetic stirring at 90 °C were required to reach a maximum yield of 82%. The methodology was then extended to the synthesis of various substituted benzodiazepines to produce excellent yields. The authors proposed a mechanism involving the assistance of the acidic protons of DACO-diacetate, which increased the electrophilic character of benzadehyde’s carbonyl (Scheme 18).

#### 2.4.2. Ultrasound-Assisted Synthesis of Biologically Active Heterocycles Under Solventless and Catalyst-Free Conditions

**Scheme 19:**
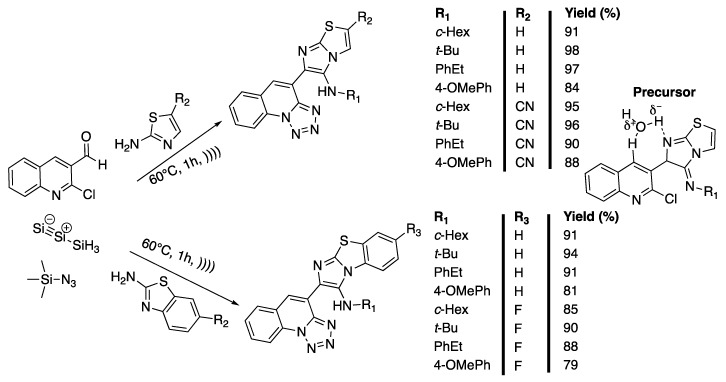
Catalyst- and solvent-free one-pot synthesis of imidazo[2,1-b]thiazoles and benzo[d]imidazo[2,1-b]thiazoles under ultrasonic irradiation using f = 42 kHz. Adapted from Claudio-Catalań et al. [106].

Tetrazolo[1,5-a]quinolines and imidazo[2,1-b]thiazoles are important molecules that exhibit a wide variety of biological activities and have potential uses as antidiabetics [100], antifungals [101], and antibiotics [102], and against age-related diseases [103]. Tetrazolo[1,5-a]quinolones [104] and imidazo[2,1-b]thiazoles [101,105] are traditionally synthesized under harsh conditions requiring toxic chemicals, high temperatures, or expensive catalysts, and have a limited substrate scope. In 2018, Claudio-Catalań et al. [106] developed a sono-assisted, solvent- and catalyst-free, one-pot green synthesis of bis-heterocycles containing imidazo[2,1-b]thiazole or benzo[d]imidazo[2,1-b]thiazole. The 2-chloro-3-formyl-quinoline, 2-aminothiazole, cyclohexyl isocyanide, and trimethylsylilazide (TMSN3) were first selected as reactives of the model reaction performed to find the optimum conditions of the Groebke−Blackburn−Bienayme (GBBR)/SNAr/ring-chain azido-tautomerization involved in the synthetic strategy. The efficiency of the solvent was studied and no reaction occurred in water in 24 h at 25 °C, whereas traces were observed in ethanol under the same conditions. No better results were obtained in 12 h of reaction in water when increasing the temperature to 80 °C, but 27% of the product were isolated in the same conditions when using ethanol as a solvent. Traces of the product were obtained in 2 h of ultrasonic irradiation at 60 °C in water versus 15% in ethanol under the same conditions. Finally, 91% of the product was obtained under neat conditions in 1 h of ultrasonic irradiation at 60 °C. The authors then explored the scope of the methodology by varying 2-aminoazines and isocyanides, keeping the 2-chloro-3-formyl-quinoline constant. Imidazo[2,1-b]thiazoles were obtained with good to excellent yields (84% to 98%). Furthermore, benzo[d]imidazo[2,1-b]thiazoles that were synthesized with 79% to 94% yields (Scheme 19). The authors then proposed a mechanism involving the formation of a precursor of the GBBR product via a non-concerted route from the imine that was obtained via condensation of 2-aminothiazole with aldehyde. This hypothesis was confirmed using DFT (Density Functional Theory) computational studies. Unfortunately, the compounds tested did not show strong anti-amebic and antibacterial activities.

Quinazolines and quinazolinones constitute a class of biologically active nitrogen heterocyclic compounds with high therapeutic potential. Indeed, they possess a wide range of biological activities, such as antimalarial, anticancer, antimicrobial, sedative, anesthetic, anti-inflammatory, antileishmanial, antidepressant, and many others [107,108]. Several drugs contain a quinazoline or quinazolinone ring, such as dacomitinib, alfusozine, bunazosin, Gefitinib, proquazone, letermovir, and afatinib [107].

**Scheme 20:**
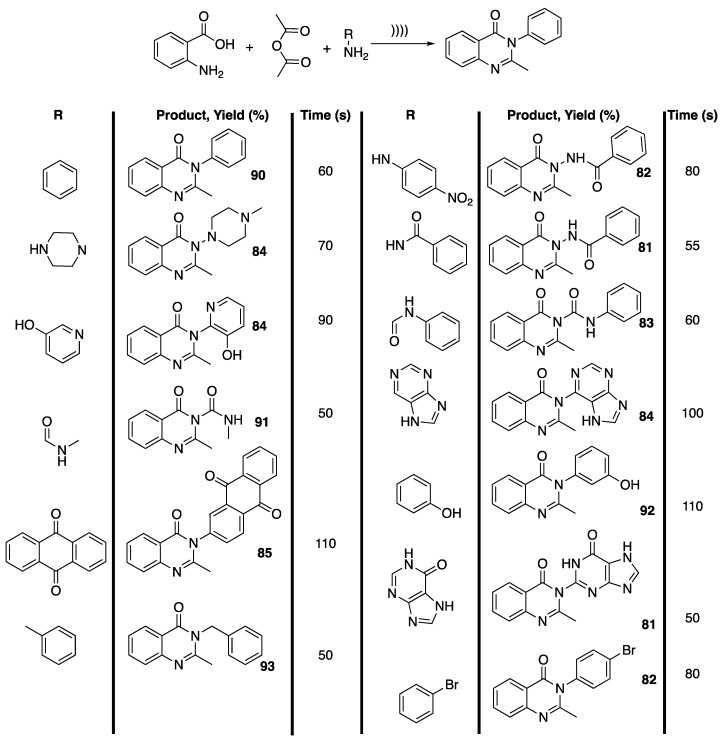
One-pot solvent and catalyst-free synthesis of 3-substituted 2-methyl quinazoline-4-(*3H*)-ones under ultrasonic irradiation. The ultrasonic probe used f = 24 kHz, P = 80% × P_output_ = 600 W, and a pulse cycle of 1. Adapted from Purkhosrowa et al. [109].

In this context, Purkhosrowa et al. [109] reported on the one-pot, solvent- and catalyst-free synthesis of new quinolone derivatives under ultrasonic irradiation and their vasorelaxant activity. To this end, the authors first reacted anthranilic acid, acetic anhydride, and anilines under ultrasonic irradiation to finalize the reaction conditions. Interestingly, under ultrasonic irradiation, 90% of condensation product were obtained in only 1 min compared to 72% with tungstophosphoric acid as a catalyst and in 40 min in refluxing ethanol. A series of primary amines were thus reacted, leading to 81% to 93% of condensation product in 50 to 110 s of reaction, depending on the nucleophilicity of the primary amines (Scheme 20). Unfortunately, a pharmacological study revealed that all the newly synthesized quinazoline derivatives exhibited vasorelaxant activity on isolated thoracic rat aorta.

**Scheme 21:**
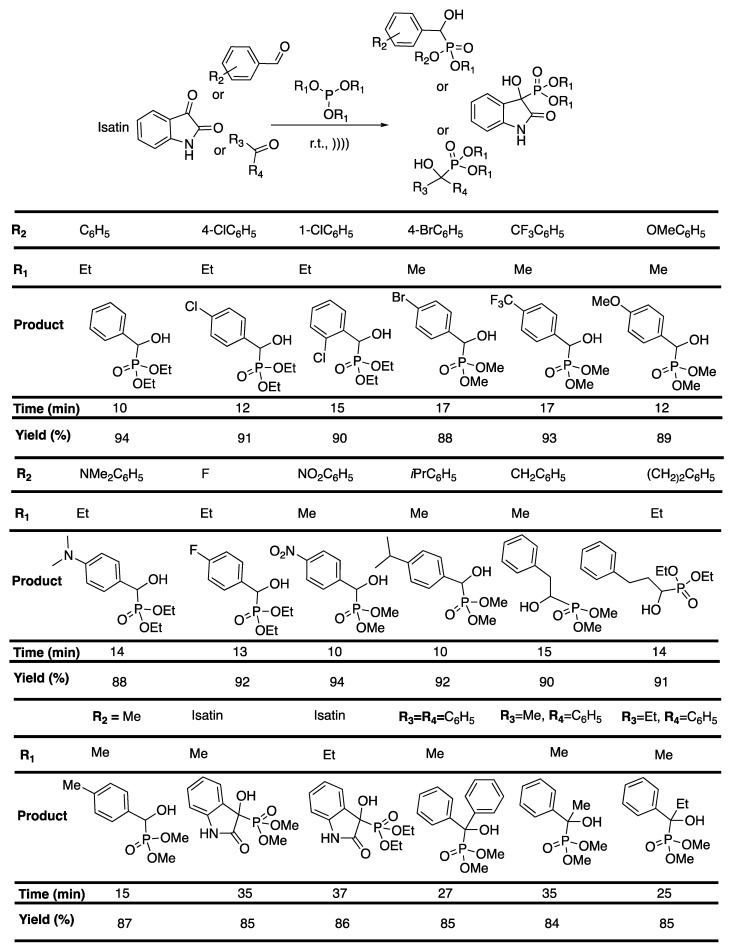
Ultrasound-assisted synthesis of a-hydroxyphosphonates at room temperature under solvent- and catalyst-free conditions. The ultrasonic bath used f = 40 kHz and P_input_ = 250 W. Adapted from Bouzina et al. [115].

α-Hydroxyphosphonates are known for their potential effect as enzyme inhibitors [110], antibiotics [111], and antibacterial or antifungal [112] agents. Unfortunately, most of the methodologies describe the use of complex and/or expensive catalysts [113], harsh acidic or basic conditions, and high temperature or long reaction duration [114]. Bouzina et al. [115] reported a straightforward, solvent- and catalyst-free, ultrasound-assisted protocol for the synthesis of α-hydroxyphosphonates. Various aromatic aldehydes were reacted with trialkylphosphite, leading to the corresponding α-hydroxyphosphonates in a short time with an excellent yield. The reaction was then extended to two derivatives of isatin, and then to three ketones, leading to a good yield in 35 to 37 min for isatin derivatives, and 25 to 35 min for ketones (Scheme 21). Pleasantly, this protocol was found to be easily scalable.

## 3. Sustainable Catalytic Transformations

Catalytic transformations hold a central position in both laboratory and industrial processes. Besides conventional catalytic activation systems, ultrasound allows for working in mild reaction conditions (temperature, short reaction time, etc.) and allows for generating highly reactive species that are usually difficult to generate in regular conditions (photolysis, pyrolysis, etc.). In heterogeneous catalysis, ultrasonic irradiation can be effective during three different stages: i) the preparation of supported catalysts, ii) the activation of the catalyst, and iii) the enhancement of the reaction rate. Ultrasound activation finds a role in many kinds of catalytic transformation, such as organometallic catalysis, acid catalyzed reactions, or enzymatic catalysis.

### 3.1. Green Oxidations

**Scheme 22:**
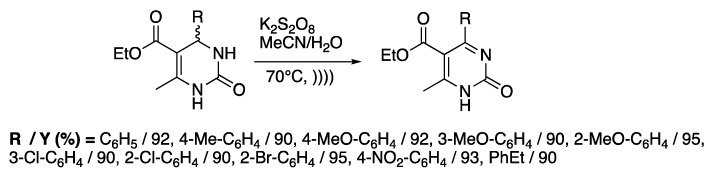
Sono-thermal oxidation of 3,4-dihydropyrimidin-2(1H)-ones. The immersion horn used f = 24 kHz, I_input_ = 460 W·cm^−2^. Adapted from Memarian and Farhadi [117].

Dihydropyrimidinones represent important classes of heteroaromatic compounds with antiviral, antibacterial, or antitumoral activities [116]. Even if 1,4-dihydropyridines can be easily oxidized by a wide range of mild oxidants, harsher conditions must be used for the aromatization of 3,4-dihydropyrimidines. Through the combination of ultrasound and potassium peroxydosulfate (PPS), Memarian and Farhadi [117] reported the sono-thermal oxidation of a variety of 3,4-dihydropyrimidines in few minutes at 70 °C in a mixture of water and acetonitrile with good yields. When the reaction was run at room temperature, no conversion was detected under silent conditions, whereas a slow formation of the desired compound was observed under ultrasonic irradiation. Under thermal activation only (70 °C), a full conversion of the starting material was observed after 80 min, whereas only 11 minutes was required using sono-thermal activation (Scheme 22). In fact, the increase of the temperature led to an activation of the oxidizing reagent through breaking the O-O bonds. Ultrasonic irradiation drastically improves the reaction rate; authors explained this phenomenon in terms of the perfect homogeneity of the medium, an efficient mass transfer, a swift temperature increase, and an effective O-O cleavage under ultrasonic irradiation.

**Scheme 23:**
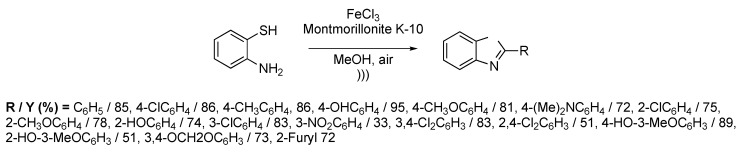
Synthesis of 2-substituted benzothiazoles catalyzed using FeCl_3_/Montmorillonite. The ultrasonic bath used f = 40 kHz and P_input_ = 250 W. Adapted from Chen et al. [118].

Among various heterocycles of medicinal interest, benzothiazole derivatives find applications in antitumoral, antiviral, or antitumoral treatments. Common methods to get 2-substituted benzothiazoles are usually based on the condensation of *o*-aminothiophenols with functionalized electrophiles like nitriles, carboxylic acids, esters, or acyl chlorides. Another strategy resides in a two-step process with aldehyde condensation followed by an oxidation. Nevertheless, this kind of synthesis suffers from long reaction times and expensive reactants. Chen et al. [118] described the one-pot synthesis of 2-substituted benzothiazoles under ultrasonic irradiation using the combination of FeCl_3_ and Montmorillonite K-10. The catalyst was prepared prior to the reaction by mixing FeCl_3_ and Montmorillonite in methanol at room temperature for 1 h. Benzothiazole derivatives were obtained by reacting *o*-aminothiophenol with one equivalent of aromatic aldehyde in the presence of 10 mol% of the FeCl_3_/Montmorillonite catalyst. The methodology was extendable to a wide range of substituted aromatic aldehydes (20 examples) (Scheme 23), giving 2-substituted benzothiazoles in moderate to good yields (51%–95%). The optimization of the reaction has shown that the best yields were obtained in polar protic solvents, such as methanol, but there is no real influence of the temperature in the reaction. Ultrasonic irradiation allowed for working in mild conditions (25–30 °C) with relatively short reaction times (0.7 to 1.2 h). Furthermore, the catalytic system FeCl_3_/Montmorillonite can be regenerated and re-used twice without a noticeable loss of the reaction yield. 

### 3.2. Epoxidation of Alkenes

**Scheme 24:**
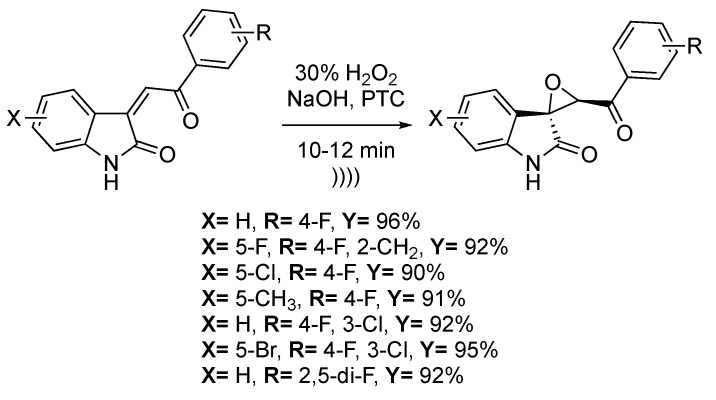
Stereoselective epoxidation of 3-aroylmethylene indole-2-one. The ultrasonic tip probe used f = 40 kHz and P_input_ = 750 W. Adapted from Dandia et al. [122].

Spiro-epoxyoxindoles have shown antitubercular, anticancer, and antifungal properties [119,120,121]. Dandia et al. [122] reported the stereoselective epoxidation of 3-aroylmethylene indole-2-one in biphasic conditions under ultrasonic irradiation (Scheme 24). Spiro[indole-3,2′-oxirane]-3′-benzoyl-2(1H)-ones derivatives were obtained with high yields (91%–96%) in one hour using hydrogen peroxide in a basic medium and with cetyltrimethylammonium bromide as a phase transfer catalyst. The three main parameters of this reaction are the phase transfer catalyst and base amounts and the temperature. The best yields were obtained for a 1:1.5:1 ratio between the substrate: H_2_O_2_: NaOH in the presence of 5 mol% of a phase transfer catalyst. Furthermore, a great diastereoselectivity of 95:5 was observed for the trans configuration.

**Scheme 25:**
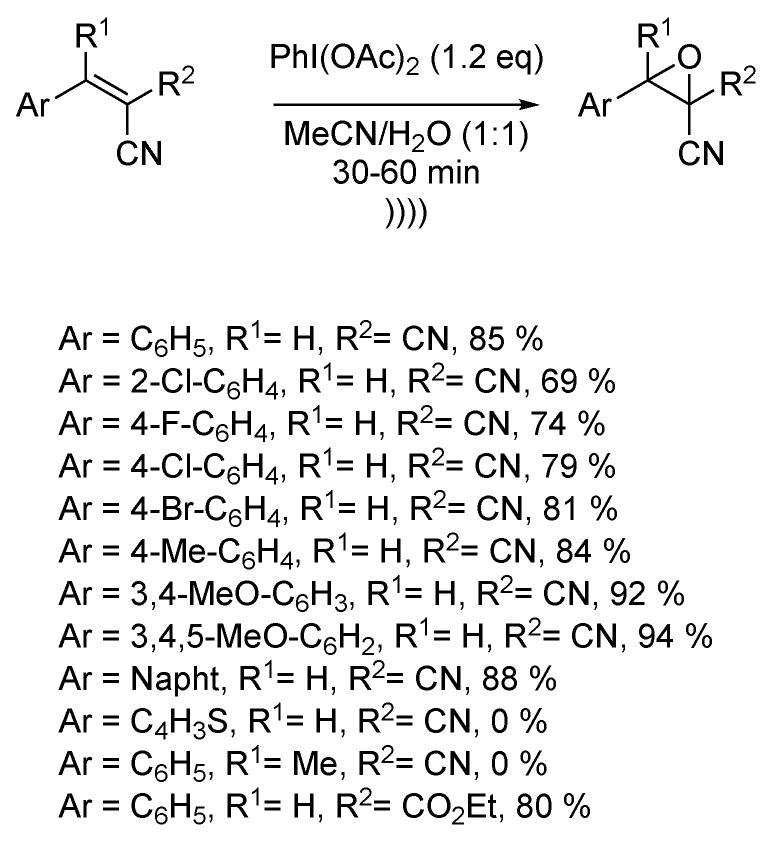
Epoxidation of β-cyanoalkenes under ultrasound. The ultrasonic bath used f = 40 kHz and P_input_ = 50 W. Adapted from Singh et al. [126].

Cyanoepoxides are essential building blocks for the synthesis of many biologically active heterocyclic compounds [123,124,125]. Recently, Singh et al. described the ultrasound-assisted synthesis of several β-cyanoepoxides via epoxidation of corresponding β-cyanostyrenes [126]. The reaction takes place in a 1:1 mixture of water and acetonitrile, using the hypervalent iodide phenyliodo diacetate (PIDA) as an oxidant. The reaction process is compatible with electron-giving and electron-withdrawing substituents on the aromatic ring of the styrene moiety. A wide range of aromatic derivatives was synthesized, even if the epoxidation process could not happen with tetra-substituted alkenes or heteroaromatic thiophene. The employment of ultrasound decreased the reaction time from several hours to a few minutes. Furthermore, the reaction could be carried out at room temperature compared to 80 °C under regular conditions (Scheme 25). By way of explanation, the authors supposed the formation of an iodonium bridge as a reaction intermediate. Ultrasound would favor the ring opening and the concomitant nucleophilic attack of a molecule of water to yield the final epoxide.

### 3.3. Enzymatic Transformations

**Scheme 26:**
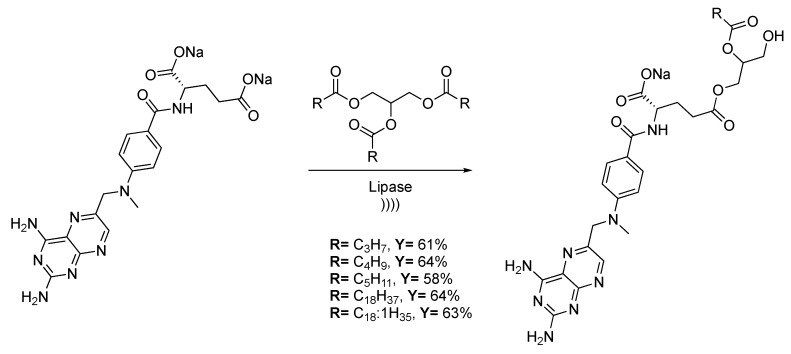
Reactional scheme of methotrexate-acylglycerols conjuguate synthesis. The ultrasonic bath used f = 40 kHz and P_input_ = 250 W. Adapted from Noro et al. [128].

Enzymatic catalysis is part of a growing interest over the last decade, thanks to high selectivity in enantio-, chimio-, and regio-selective chemical transformations. Ultrasound improves enzymatic reactions via the enhancement of substrate dissolution and mass transfer, which induce the conformation changes of proteins. Methotrexate is used in lymphocytic leukemia, lymphomas, and for some solid tumor treatments [127]. Thanks to the triacylglycerol moiety, the toxicity of the pro-drugs decreases compared to the usual methotrexate-glyceride pro-drugs. Recently, Noro et al. [128] described the transesterification of methotrexate with several triacylglycerol derivatives. The reaction is carried out in water and catalyzed by *Candida antartica Lipase B* supported on glycidyl methacrylateter-divinylbenzene-ter-ethylene glycol dimethacrylate (Scheme 26). The transesterification process occurs in three steps: First, the triacylglycerol is hydrolyzed twice by the enzyme to generate an intermediate at the active center of the enzyme, with only one available side chain. Then, the methotrexate binds the enzyme at the active site, and finally, a nucleophilic attack of the glycerol unit leads to the formation of the final conjugate. As the methotrexate is poorly soluble in almost all solvents, ultrasound allows for obtaining high conversion rates thanks to a strong emulsification of the medium.

**Scheme 27:**
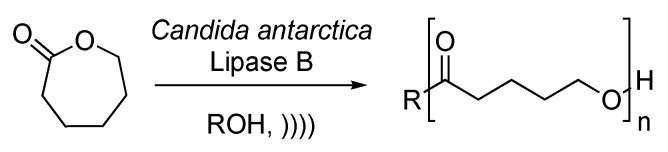
Synthesis of poly-6-hydroxyhexanoate under enzymatic catalysis. The ultrasonic bath used f = 45 kHz and P_input_ = 120 W. Adapted from Gumel et al. [129].

Gumel et al. reported a ring opening followed by polymerization of caprolactone, catalyzed by *Candida antarctica Lipase B* [129]. The polymerization leads to the formation of poly-6-hydroxyhexanoate, which finds many applications in biomedical industries thanks to its biocompatibility, biodegradability, and attractive mechanical properties. The reaction is carried out in an ionic solvent under ultrasonic irradiation (Scheme 27). The first step of the process is the formation of an acyl-enzyme complex. The nucleophilic attack of the complex by a molecule of water leads to the formation of ϖ-hydroxycarboxylic acid. The resulting acyl-enzyme complex can then undergo further nucleophilic attack with the terminal hydroxyl function of a previously formed ϖ-hydroxycarboxylic acid. Ultrasound enhances the molecular weight and crystallinity of the final polymer and decreases its polydispersity index. Furthermore, under silent conditions, the conversion drops drastically due to a loss of the enzyme activity and an accumulation of water produced during the reaction process.

### 3.4. Green Reductions

**Scheme 28:**
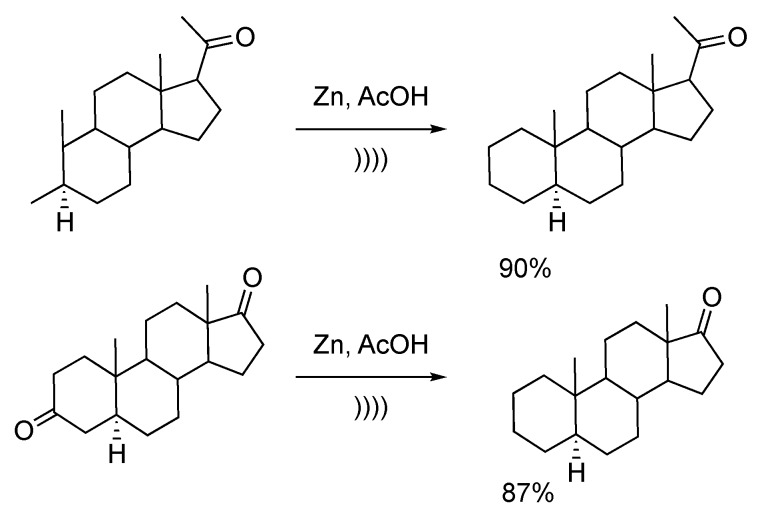
Clemmensen-type reduction of steroid derivatives using an ultrasonic bath. Adapted from Yamamura [131].

Ultrasonic irradiation enhances the catalytic activity of zinc powder via deep modification of the particles’ morphology. Salvador et al. [130] described the Clemmensen-like reduction of a series of steroid-based ketones by using the combination of ultrasound and zinc dust. The reduction occurred by mixing the keto-compounds with a large excess of zinc dust for 15 minutes between 15 and 25 °C in pure AcOH or AcOH/H_2_O media. The use of ultrasound clearly enhances the reaction rate and allows for working under mild conditions and avoiding the formation of by-products. Interestingly, for polyketo derivatives, the reduction is specific to the 3-position of the steroid scaffold. In fact, the steric hindrance around other positions (position 17 or 20 on Scheme 28) combined with mild conditions, explain the lack of reactivity with these other positions [131].

**Scheme 29:**
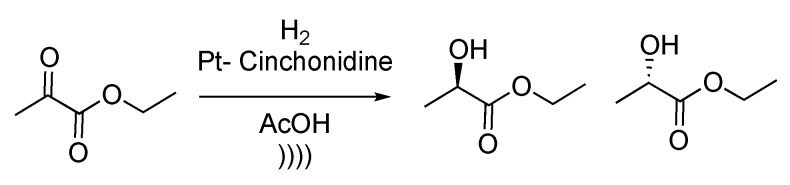
Enantioselective reduction of ethyl pyruvate. The ultrasonic bath used f = 20 kHz and P_input_ = 30 W. Adapted from Török et al. [132].

An enantioselective ethyl pyruvate reduction was then reported by Török et al. [132]. The reduction was performed using a SiO_2_- or Al_2_O_3_-supported Pt-cinchonidine catalytic system. Reactions were carried out in acetic acid with 3 to 5 mol% of catalyst under hydrogen pressure (Scheme 29). Enantioenriched ethyl lactate isomers were obtained up to 97% ee. Authors reported that there is a direct effect between ultrasonic irradiation of the catalyst and final enantiomeric excess because of the strong modification of the particles’ morphology under ultrasound. Indeed, after 30 min of sonication, the particles reached the optimal average size in terms of enantiospecificity of the reaction. The authors compared the effect of two different ways of sonication, using a bath or a probe. The conclusion of this study is that for a heterogeneous reaction, better results were obtained using a sonication bath, whereas a probe was more efficient for homogeneous reactions.

**Scheme 30:**
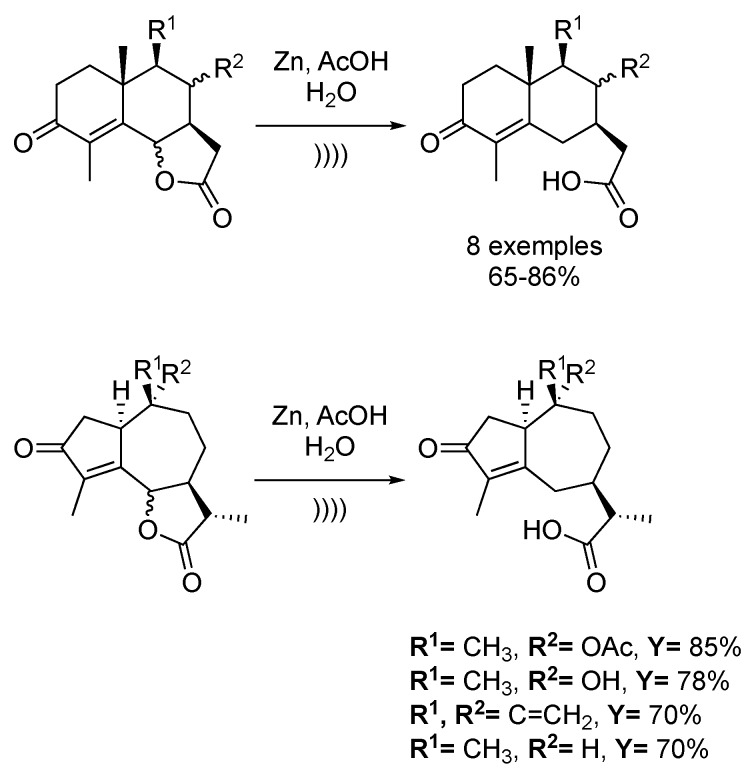
Total synthesis of 1α,7α,10αH-guaian-4,11-dien-3-one. The ultrasonic bath used f = 40 kHz and P_input_ = 100 W. Adapted from Blay et al. [133].

Sesquiterpene lactone derivatives are interesting building blocks for the total synthesis of natural products. Pedro et al. [133] reported the ultrasound-assisted reductive cleavage of several γ-enonelactones (Scheme 30). The first investigation using aluminium nickel chloride or zinc nickel chloride as heterogeneous catalysts did not yield the expected products of hydrogenolysis. However, when the reactions were carried out in a mixture of acetic acid and water in the presence of an excess of zinc dust under ultrasonic irradiation, hydrogenolysis products were obtained in moderate to good yields. Compared to usual methods where sesquiterpene acids are prepared in a two-step process via epimerization of the *trans*-lactone to the *cis*-lactone, this methodology allows one to work directly with the *trans* isomer.

**Scheme 31:**
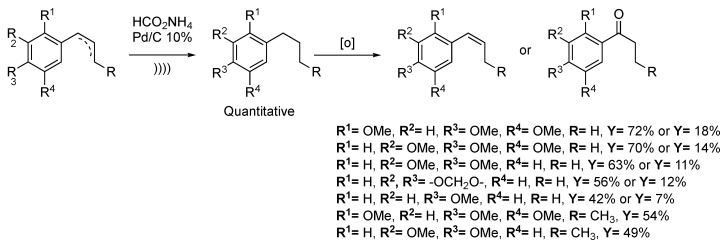
Synthesis of arylalkenes and arylalkanones. The ultrasonic bath used f = 20 kHz and P_input_ = 400 W. Adapted from Joshi et al. [137].

Natural arylalkanoids have proven biological activities in neuroleptic, anti-ulcerogenic, anti-inflammatory, or anti-fungal treatments [134,135,136]. Joshi et al. [137] described the two-step synthesis of arylalkenes and arylalkanones, starting from abundantly available but toxic (*Z*)-1-(2′,4′,5′-trimethoxyphenyl)prop-1-ene. The first step consists in the hydrogenation of the alkene moiety using 10% Pd/C as a catalyst and ammonium formate as a proton source under ultrasonic irradiation. The desired compounds are obtained in a quantitative yield in 30 min when 10–12 h are required with conventional methods. The methodology was applied to 10 derivatives and yielded the reduced compounds quantitatively each time. Depending on dry or wet conditions, the oxidation step allows for obtaining arylalkene or arylalkanone selectively (Scheme 31).

Ultrasound is also known to be effective in homogeneous catalysis. In particular, the ultrasonic irradiation of CHCl_3_ leads to the production of a small amount of HCl (see Equation (1)) [138,139,140]. The same reactivity was observed with the sonication of a mixture of CCl_4_ and aliphatic alcohol (see Equation (2)). Indeed, the low vapor pressure of CCl_4_ induces a violent collapse of cavitation bubbles and favors the production of HCl [141].

*n* CHCl_3_ ⟶ HCl + CCl_4_ + CH_2_Cl_2_ + C_2_HCl_3_ (1)

Equation (1): HCl production via the sonication of CHCl_3_. The ultrasonic bath used f = 20 kHz and P_input_ = 50 W. Adapted from Suslick [141].

*n* CHCl_4_ + ROH ⟶ HCl + Cl_2_ + ROCl + C_2_Cl_2_ (2)

Equation (2): HCl production via the sonication of a CCl_4_/alcohol mixture. The ultrasonic bath used f = 20 kHz and P_input_ = 50 W. Adapted from Suslick [141].

**Scheme 32:**
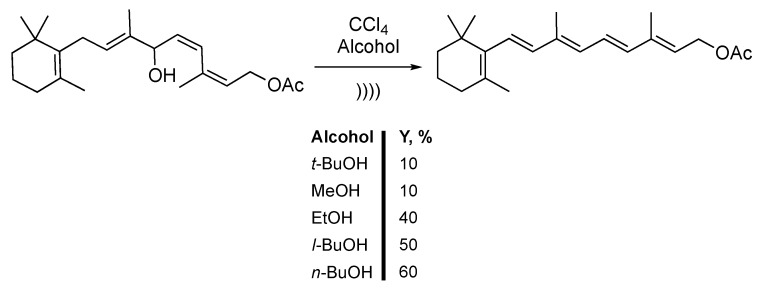
Acid-catalyzed dehydration-isomerization of hydroxenine monoacetate. No information concerning the apparatus were given. Adapted from Bonrath et al. [142,143] and Aquino et al. [144].

Using the properties of the sonication of chlorinated solvents, Bonrath et al. [142,143] and Aquino et al. [144] described the synthesis of vitamin A acetate via the dehydration of hydroxenin monoacetate. The reaction was carried out in a mixture of CCl_4_ and an aliphatic alcohol (Scheme 32). After protonation of the substrate, dehydration of the allylic alcohol leads to the formation of a carbocation. Interestingly, higher yields were obtained under mild conditions at 0 °C and with low ultrasonic power. In fact, low temperatures induce a lowering of the vapor pressure [145]. The authors claimed that after 6 hours of reaction, less than 10% yield was obtained by using *tert*-butanol or methanol. Using ethanol improves the yield to 40%. Finally, *iso*-butanol and *n*-butanol leads to 50% and 60% yields, respectively. This methodology allows for accessing vitamin A acetate in an all *E* configurations of double bonds.

Tryptamine, which is found in fungi, plants, and animals [146] are naturally occurring indole alkaloids that exhibit a broad range of medicinal and biological activities [147]. Thus, α-methyltryptamine may act as an antidepressant, a powerful psychedelic drug, a stimulant, and a monoamine oxidase inhibitor [148]. Similarly, 5-carboxamidotryptamine presents a powerful vasodilator effect [149]. Many marketed drugs contain an indole ring [150] and the development of powerful but sustainable methodologies for their synthesis is therefore of great interest.

**Scheme 33:**
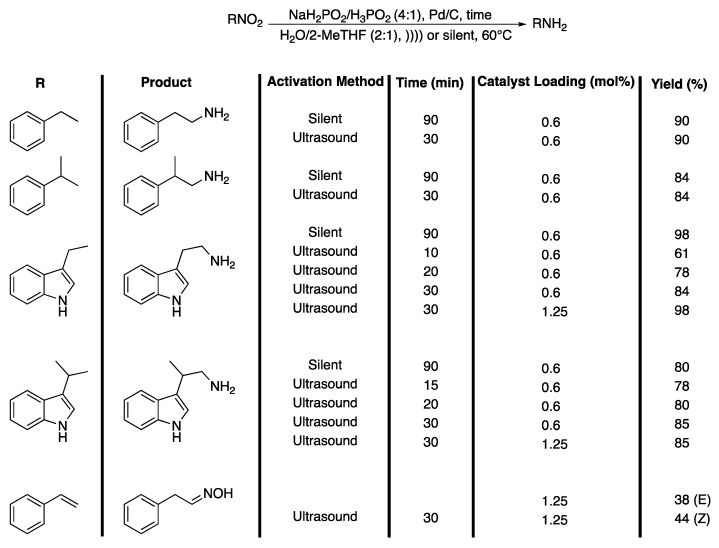
Reduction of β-nitro-aromatic derivatives. )))) = ultrasonic irradiation, sonotrode used f = 20 kHz and P_acoustic_ = 3.94 W. Adapted from Letort et al. [151].

In that context, Letort et al. [151] described an efficient process for the synthesis of tryptamine and amine derivatives from their nitro precursor under ultrasonic irradiation in water. They studied the reduction of 2-nitroethylbenzene, the model substrate, to 2-aminoethylbenzene, in terms of the solvent effect, NaH_2_PO_2_/H_3_PO_2_ ratio, temperature, Pd/C catalyst loading, and the effect of the ultrasound. In order to differentiate the effects of the ultrasound from those of temperature, the reactions were thermostatically controlled. In 16 h of magnetic stirring at 60 °C, the reaction was shown slightly to be more efficient in the (2:1) H_2_0/2-MeTHF mixture than in water with a 4:1 ratio of NaH_2_PO_2_/H_3_PO_2_ and yielded 80% of 2-phenylethan-1-amine. The authors explained the role of Pd/C and the mechanism of reduction by hypophosphites that are strong reducing agents registered by REACH as non-hazardous substances for human beings and for the environment. In order to increase the rate of the reaction while increasing the yield in amines, the reaction was then performed under ultrasonic irradiation and the results obtained were compared to those obtained under silent conditions. Ultrasonic irradiation was shown to speed up the reaction, especially in a mixture of H_2_O/Me-THF (2:1), and a maximum yield of 92% of 2-phenylethan-1-amine was obtained at 60 °C in only 15 min under ultrasonic irradiation compared to 90 min under silent conditions. Moreover, this effect is much more obvious in water as a solvent. Indeed, whereas 90 min are required to obtain 35% of amine under silent conditions, double this amount is obtained under ultrasonic irradiation. In water and under ultrasonic irradiation, a maximum yield of 90% is obtained in 15 min when the temperature is increased to 70 °C. In order to discriminate which of the physical or chemical effects of ultrasound are predominant, the authors then carried out the reaction using a vibroacoustic mixer, giving rise to hydrodynamic cavitation and leading only to physical effects. It was clearly shown that the predominant effects of ultrasonic irradiation on the reduction reaction were of a physical nature. The reaction was classified as a type II reaction termed “false sonochemistry.” The methodology scope was then extended to other nitro compounds and tryptamine derivatives leading to a good yield in reduction compounds in a shorter time under ultrasonic irradiation (Scheme 33).

## 4. Conclusions

This review aimed to present a comprehensive view of the use of ultrasound for a more sustainable synthesis of bioactive molecules through the presentation of the latest most representative studies published in the literature. Unfortunately, little information on important parameters, such as acoustic power, reactor geometry, etc., are generally given, jeopardizing the possibility of rational explanations regarding the impact of ultrasound on the reaction, the mechanism, or on physical aspects of the progress of reactions. Thus, no in-depth mechanistic study of the reactions is given under ultrasonic irradiation, and that role is generally not fully explained. These kinds of information would help chemists to gain valuable insights into the potential of ultrasound for organic chemistry, and especially drug synthesis, thus providing new directions for future investigations. Nevertheless, the diverse reactions described show obvious improvements under ultrasonic irradiation, highlighting the potential of this non-conventional tool in helping toe make the synthesis of bioactive molecules more sustainable.

## Figures and Tables

**Table 1:** Determination of the reaction conditions for the synthesis of rhodanine derivatives using model reagents ^1^. Adapted from Rostamnia and Lamei [44].

Solvent	Activation Method	Yield (%)
H_2_O	Ultrasound	94
H_2_O	Silent	<10
EtOH	Ultrasound	90
EtOH	Silent	<10
EtOH-H_2_O (1:1)	Ultrasound	93
EtOH-H_2_O (1:1)	Silent	<10
THF	Ultrasound	91
THF	Silent	<10
MeCN	Ultrasound	92
MeCN	Silent	<10

^1^ T = 34–36 °C, t = 3 min. Yield of isolated product was based on the starting alkyne, where solvent = 40 mL.
